# Albuterol enantiomer levels, lung function and QTc interval in patients with acute severe asthma and COPD in the emergency department

**DOI:** 10.1186/1865-1380-4-30

**Published:** 2011-06-15

**Authors:** Kwang Choon Yee, Glenn A Jacobson, Richard Wood-Baker, E Haydn Walters

**Affiliations:** 1 School of Pharmacy, University of Tasmania, Hobart, Tasmania, Australia; 2 Menzies Research Institute, University of Tasmania and Department of Respiratory Medicine, Royal Hobart Hospital, Hobart, Tasmania, Australia

## Abstract

**Background:**

This observational study was designed to investigate plasma levels of albuterol enantiomers among patients with acute severe asthma or COPD presenting to the emergency department, and the relationship with extra-pulmonary cardiac effects (QTc interval) and lung function. Recent reviews have raised concerns about the safety of using large doses of β_2_-agonists, especially in patients with underlying cardiovascular comorbidity. It has been demonstrated that significant extrapulmonary effects can be observed in subjects given nebulised (R/S)-albuterol at a dose of as little as 6.5 mg.

**Methods:**

Blood samples were collected and plasma/serum levels of (R)- and (S)-albuterol enantiomers were determined by LC-MS and LC-MS/MS assay. Extra-pulmonary effects measured at presentation included ECG measurements, serum potassium level and blood sugar level, which were collected from the hospital medical records.

**Results:**

High plasma levels of both enantiomers were observed in some individuals, with median (range) concentrations of 8.2 (0.6-24.8) and 20.6 (0.5-57.3) ng/mL for (R)- and (S)- albuterol respectively among acute asthma subjects, and 2.1 (0.0-16.7) to 4.1 (0.0-36.1) ng/mL for (R)- and (S)- albuterol respectively among COPD subjects. Levels were not associated with an improvement in lung function or adverse cardiac effects (prolonged QTc interval).

**Conclusions:**

High plasma concentrations of albuterol were observed in both asthma and COPD patients presenting to the emergency department. Extra-pulmonary cardiac adverse effects (prolonged QTC interval) were not associated with the plasma level of (R)- or (S)-albuterol when administered by inhaler in the emergency department setting. Long-term effect(s) of continuous high circulating albuterol enantiomer concentrations remain unknown, and further investigations are required.

## Background

Albuterol (salbutamol), a β_2_-agonist, plays an important role in emergency medicine and is the first line medication for relief of shortness of breath during acute asthma exacerbations. Albuterol is also used on a regular basis for the management of chronic obstructive pulmonary disease (COPD), both during stable periods and acute exacerbations [1-3]. Many recent studies and guidelines have indicated that the use of short-acting β_2_-agonists on a regular basis will not improve asthma control, and may even cause deterioration [4-6]. However, regular use of short-acting β_2_-agonists such as albuterol is still very common for the management of COPD [1-3].

Albuterol is a chiral compound consisting of (R)- and (S)- enantiomers, and is most commonly administered as a 1:1 racemic mixture (*rac-*). The therapeutic effect of albuterol is supposedly delivered by the (R)-enantiomer [7]. However, (R)- and (S)- albuterol have been found to exhibit different pharmacokinetic properties, where (S)-albuterol has greater bioavailability and a longer half-life than (R)-albuterol [8,9].

These differences in the pharmacokinetics of albuterol enantiomers can contribute to the accumulation of (S)-albuterol after repeated dosing [8,10]. Some studies have claimed that (S)-albuterol is not inert, but rather has detrimental physiological effects, including pro-inflammatory and pro-constriction effects [11,12], increases airway responsiveness [13,14] or acts as a functional antagonist [15]. Potential adverse effects of (S)-albuterol have also been suspected since studies found that pure (R)-albuterol is superior in treatment outcomes compared to the equivalent dose of *rac*-albuterol [16-18]. However, these findings are usually difficult to interpret and are often not translated into clinical studies that compare the therapeutic outcome [19-22]. There are a number of studies indicating that both the immediate therapeutic effects and immediate adverse effects of *rac*-albuterol are delivered solely by (R)-albuterol [9,23,24]. The weight of evidence to date suggests that (S)-albuterol is inert, but the effects of high levels of (S)-albuterol remain unclear [19,22].

Most of the pharmacokinetic and pharmacodynamic studies of albuterol have been performed on healthy, mildly asthmatic patients, within the generally recommended dose [8,9,15,23]. However, patients presenting to the emergency department with exacerbations of asthma and/or COPD are usually heavily reliant on short-acting β_2-_agonists for symptom relief prior to presentation and would be expected to use much higher doses of albuterol. A study has shown that patients who have died from asthma have up to 2.5-fold higher plasma albuterol levels than asthma patients using albuterol at the emergency department [25]. In addition, studies have shown that the significant extrapulmonary effects of inhaled albuterol, which include increased heart rate [9,24,26,27], increased QT interval [26] and decreased plasma potassium level [9,24,26,28] can all occur within the maximum recommended dose. It has been suggested that the presence of β_2_-agonists can aggravate the risk of these cardiovascular events, in particular among individuals who have long-term exposure to accumulated doses of β_2_-agonist [27,29].

Our preliminary investigations in emergency department presentations have revealed relatively high plasma levels in acute severe asthma patients, with an up to five-fold difference in concentrations of (R)- and (S)-albuterol [30]. The objective of this study was to observe the relationship between (R)- and (S)-albuterol levels and lung function measures, as well as potential extrapulmonary adverse effects, in presentations of acute disease exacerbation seen in a typical emergency department setting.

## Method

### Study design

The study was observational in design and conducted in two separate phases. The study was designed to observe the relationship between albuterol enantiomer levels and lung function measures and potential extrapulmonary adverse effects among patients presenting with exacerbation of asthma and COPD respectively.

The study was conducted at the Department of Emergency Medicine (DEM), Royal Hobart Hospital (RHH), Tasmania, Australia. The study was approved by the State Human Research Ethics Committee in compliance with the Helsinki Declaration, and written informed consent was obtained from all subjects prior to the investigation.

### Acute asthma study subjects

Potential subjects of the study were patients who presented to the DEM with an acute exacerbation of asthma. The inclusion criteria were adult patients, aged between 18 and 65 years, and self-reported *rac-*albuterol utilisation within 24 h prior to presentation. Recruitment was convenience sampling in nature and was conducted in two phases over a total period of 18 months.

Patients who had presented to the emergency department for over 12 h prior before blood sampling were excluded. Moderate to severe asthma exacerbation was diagnosed by independent emergency physicians, in accordance with the National Asthma Council Australia (NAC) guidelines [31].

### Acute asthma sample and data collection

Blood samples (10 mL) were collected from each subject in potassium EDTA tubes by medical or nursing staff at the DEM. The blood sample was then centrifuged, and the plasma harvested and stored at -20°C until analysis.

History of *rac*-albuterol use by subjects within the previous 24 h was obtained from subjects by interview and from medical records. The albuterol utilisation was also converted to defined daily dose (DDD) [32], which was designed to standardise the dose between different types of formulation. One DDD of *rac*-albuterol was considered equivalent to 800 μg of *rac*-albuterol delivered by pressurised metered dose inhaler (MDI) or 10 mg delivered by nebuliser. The DDD was only used as an estimation of the number of doses of albuterol required during the asthma exacerbation (between different dosage forms), and does not represent the amount of albuterol being delivered or reflect the recommended dose.

Basic demographic information and details of medical treatment during hospital presentation and on the way to hospital were obtained from the hospital medical records. Concomitant use of other asthma medication was recorded. Clinical measures of severity and response to therapy included improvement in percent predicted PEF after 60 min and a four-point severity score, similar to the Acute Asthma Index (AAI) designed and validated by Rodrigo and Rodrigo [33]. However, a 60-min PEF was used instead of the 30-min PEF as used in the AAI, as it was more achievable by emergency department staff in our setting. Respiratory function tests were performed with a Vitalograph^® ^Compact spirometer (Buckingham, UK).

### Acute COPD study subjects

Potential subjects of this study were adult patients presenting to the DEM with exacerbation of COPD over a period of 14 months. Subjects were excluded if they did not have a routine serum sample collected within 4 h of presentation or were not admitted to the general ward after the DEM presentation. Confirmation of the diagnosis and subject recruitment (convenience sampling) were carried out at the general ward by an independent medical officer from the Department of Respiratory Medicine, RHH.

### Acute COPD sample and data collection

Serum aliquots were obtained from the remaining samples after routine blood examination was performed according to DEM procedures. Routine tests undertaken include full blood examination, electrolyte examination and ECG measurement. The Department of Clinical Chemistry (Pathology), RHH, was informed of each subject's participation, through a secure collaborative network, after written informed consent had been obtained. The remaining serum samples (collected in VACUETTE^® ^Z Serum Sep C/A tubes) were then transferred to the investigators after being kept at the Pathology Department (at 4-8°C) for 7 days as required in accordance with the RHH Pathology serum protocol. After the transfer, serum samples were stored at -20°C until analysis.

Information regarding the potential extrapulmonary adverse effects of albuterol within the 4 h of DEM presentation, including heart rate (HR), corrected QT (QTc) interval, serum potassium level and blood sugar level (BSL), was collected from hospital medical records. Demographic information and relevant medical history were extracted from medical records. Medication history prior to the ECG measurement and blood sampling, in particular medications known to affect the measurements clinically, was also recorded. ECG measurements were examined by an independent clinician to determine if the recorded QTc intervals were affected by underlying cardiac condition(s) (e.g. heart block). Subjects with a medical or medication history that could interfere with the measurement(s) were excluded from the association analysis.

### Analysis of albuterol

Albuterol enantiomer analysis was performed with a previously published method [34], modified using deuterated *rac*-albuterol (D3-*rac*-albuterol; 3-hydroxymethyl-D_2_, α-D_1_, obtained from Medical Isotopes, Inc., Pelham, NH) as internal standard. In brief, the samples were brought to room temperature, and the internal standard and ammonia buffer were added to each aliquot before solid-phase extraction and analysis by LC-MS or LC-MS/MS. The lower limit of quantification (LLoQ) was 0.156 ng/mL (from 500 μL), and reproducibility (RSD) was < 15%.

### Statistical analysis

One-way factorial ANOVA was used to assess the relationship between severity score and plasma albuterol, and Fisher's protected least significant difference (PLSD) post hoc test was used to assess any statistical significance. Linear regression was used for the relationship between continuous variables. Spearman rank correlation and Mann-Whitney tests were used to assess the relationship between the serum albuterol level and extrapulmonary effects (heart rate, QTc interval, serum potassium level and BSL), which did not exhibit Gaussian distributions. Statistical analyses were undertaken with Statview 5.0.1 (SAS Institute Australia Pty Ltd., NSW, Australia) and SPSS 15.0 for Windows (SPSS Australasia Pty. Ltd., Chatswood, NSW, Australia).

## Results

### Acute asthma

Fifteen patients were recruited for the study. Basic demographic and albuterol utilisation in the previous 24 h are summarised in Table 1. The initial baseline respiratory test (PEF) was not performed in three subjects, partly because of the severity of their symptoms, but was estimated by clinicians to be less than 25% of the predicted value.

**Table 1:** Subject's demographic and *rac*-albuterol utilisation among patients presenting to DEM with acute asthma

	Median (range) *N *= 15
Age	38 (22-65)
Gender	6 male; 9 female
Smoking history (medical record)	
Current smoker	5
Ex-smoker	2
Respiratory test, % predicted PEF (*n *= 12)	
Baseline	51 (21-69)
60-min post-initial test	60 (31-78)
Total *rac*-albuterol utilisation in preceding 24 h (DDDs)	3.0 (0.8-11.0)
Total dose delivered via MDI	1.5 (0.0-5.3)
Total dose delivered via nebuliser	2.0 (0.0-5.5)
Total dose delivered by health-care officer	1.5 (0.0-0.25)

**Figure 1:**
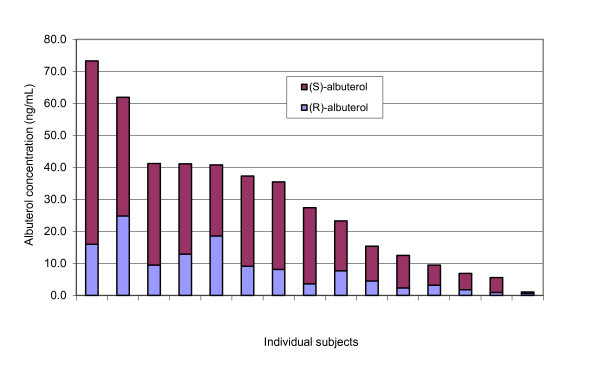
**Plasma albuterol enantiomer levels observed among subjects presenting with acute asthma exacerbation (*n *= 15)**.

Plasma albuterol enantiomer levels were measured in all subjects (Table 2 and Figure 1). There were no relationships between plasma albuterol enantiomer levels and severity or response to treatment, measured both by the four-point severity score (Table 3) and percent improvement in predicted PEF at 60 min. Patients with higher levels of 24 h *rac*-albuterol utilisation (DDDs), consistent with greater morbidity, had a lower percent predicted PEF at baseline *(r^2 ^*= 0.33, *p *= 0.03), but not a poorer response to therapy measured using the severity score [F(2,12) = 1.83, *p *= 0.20].

**Table 2:** Correlation between *rac*-albuterol dose utilisation [median (range)] and serum albuterol enantiomer levels [median (range)] among acute asthma subjects

		Albuterol utilisation	
	Serum level	**Total dose utilisation **^**a**^ 20.0 (0.6-55.0) mg	**Recorded dose utilisation **^**b**^ 15.0 (0.0-50.0) mg
(R)-albuterol	8.2 (0.6-24.8) ng/mL	*r^2 ^*= 0.22	*r^2 ^*= 0.54*
(S)-albuterol	20.6 (0.5-57.3) ng/mL	*r^2 ^*= 0.50	*r^2 ^*= 0.33
Total albuterol	28.9 (1.1-73.3) ng/mL	*r^2 ^*= 0.43	*r^2 ^*= 0.42

Two-tailed Pearson correlation test

^a^Dose administered in the preceding 24 h, including dose administered prior to the hospital presentation

^b^Dose administered by health-care officer, as recorded in hospital medical history

*p < 0.05

**Table 3:** Severity score* and albuterol plasma levels

	Median (range) plasma levels ng/mL			
**Severity score**	**Total albuterol**	**(R)-albuterol**	**(S)-albuterol**	**S:R ratio**

2 (*n *= 8)	21.5 (1.1-61.9)	4.1 (0.6-24.8)	17.4 (0.5-37.1)	3.0 (0.8-6.6)
3 (*n *= 4)	32.3 (9.5-73.3)	10.3 (3.2-16.0)	22.0 (6.3-57.3)	2.1 (2.0-3.6)
4 (*n *= 3)	35.5 (5.6-40.8)	8.1 (0.9-18.6)	22.1 (4.7-27.4)	3.4 (1.2-5.2)

*Modified from the Acute Asthma Index; AAI [36]

Neither smoking history nor the use of inhaled corticosteroids was associated with albuterol used (DDD), the percent improvement in predicted PEF at 60 min or the severity score. Subjects who had been using long-acting β_2_-agonists were found to be more likely to have used less *rac*-albuterol in the previous 24 h before presentation (*p *= 0.02).

### Acute COPD

Thirty-seven patients were recruited for the COPD phase of the study, where 25 of the subjects had a recorded medical history of a cardiovascular comorbidity (Table 4).

**Table 4:** Subject demographics and *(R/S)*-albuterol utilisation among acute COPD patients presenting to DEM

	Median (range) (*n *= 37)
Age	70 (51-85)
Gender	13 male; 24 female
Smoking history (medical record)	14
Ex-smoker	18
Comorbidity with asthma	5
Cardiovascular comorbidity	
Ischaemic heart disease	11
Heart failure	4
AF	2
Past AMI	4
Total (DDD) *rac*-albuterol delivered ^a^	0.5 (0.0-4.0)

^a^Dose delivered by health-care professionals include paramedic, doctor and nursing staff

**Figure 2:**
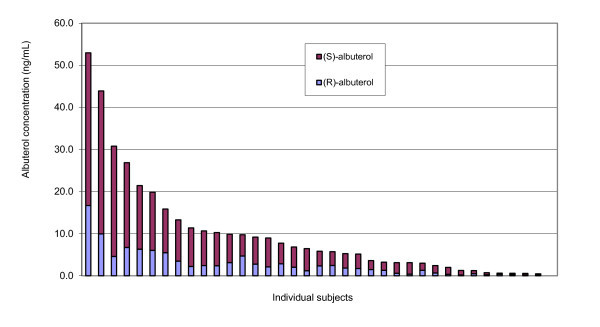
**Serum albuterol enantiomer levels observed among subjects presenting with acute exacerbation of COPD (*n *= 30)**.

Serum albuterol enantiomer levels were measured in all subjects (Table 5 and Figure 2), with a weak correlation observed between albuterol dose (mg) and total albuterol as well as (R)- and (S)-albuterol enantiomer levels.

**Table 5:** Correlation between *rac*-albuterol dose utilisation [median (range)] and serum albuterol enantiomer levels [median (range)], among acute COPD subjects

	Serum level	**Albuterol utilisation **^**a**^ 5.0 (0.0-40.0) mg
(R)-albuterol	2.1 (0.0-16.7) ng/mL	*r^2 ^*= 0.34 *
(S)-albuterol	3.5 (0.0-36.1) ng/mL	*r^2 ^*= 0.36 *
Total albuterol	5.8 (0.0-53.0) ng/mL	*r^2 ^*= 0.36 *

^a^Dose administered by health-care officer, as recorded in hospital medical history

*p < 0.01

ECG measurements were available in the medical records for 28 subjects, but 2 subjects' ECG measurements were excluded from analysis because of a concurrent digoxin toxicity and a probable atrial flutter, respectively. Six subjects (3 male and 3 female) were identified with prolonged QTc intervals ( > 440 ms and > 450 ms for males and females respectively); however, these were not associated with serum levels of total albuterol (*p *= 0.05). Results of serum albuterol levels, heart rate and QTc interval are summarised in Table 6.

**Table 6:** Mean (range) ECG measurements (HR and QTc interval), serum potassium level and BSL for each tertile of albuterol enantiomer serum level

Albuterol concentration (range)	(R)-albuterol Lower (0.0-1.2 ng/mL) Middle (1.3-2.5 ng/mL) Upper (2.8-16.7 ng/mL)	(S)-albuterol Lower (0.0-2.1 ng/mL) Middle (2.5-6.8 ng/mL) Upper (6.9-36.3 ng/mL)	Total albuterol Lower (0.0-3.1 ng/mL) Middle (3.2-9.7 ng/mL) Upper (9.9-53.0 ng/mL)
HR (/min) (*n *= 26)	89 (70-120) 103 (59-127) 109 (96-137)	88 (70-120) 102 (59-120) 109 (96-137)	89 (70-120) 102 (59-120) 109 (100-137)
QTc interval (ms) (*n *= 26)	425 (386-486) 438 (374-481) 384 (363-404)	425 (374-481) 413 (377-486) 385 (363-427)	425 (386-481) 427 (374-486) 385 (363-406)
Serum potassium level (mmol/L) (*n *= 10)	4.7 (4.4-5.3) 3.5 (-) 4.4 (4.1-5.1)	4.0 (3.9-5.3) 4.1 (3.5-5.1) 4.4 (3.9-5.0)	4.0 (3.9-5.3) 3.8 (3.5-4.1) 4.6 (3.9-5.0)
BSL (mmol/L) (*n *= 15)	6.7 (5.2-13.3) 6.0 (5.4-7.8) 7.3 (5.7-10.4)	6.7 (5.2-13.3) 6.7 (5.8-7.8) 6.2 (5.4-10.4)	6.7 (5.2-13.3) 6.1 (5.4-7.8) 6.8 (5.7-10.4)

The serum potassium levels were recorded in 34 subjects, and the BSLs were recorded in 31 subjects. However, 24 of the serum potassium results were considered inconclusive and excluded from the analysis because of the subjects' medication histories (potassium supplements, diuretics and i.v. fluid infusion) and/or faulty specimens (suspected haemolysed sample). Similarly, 17 of the BSL results were also excluded from analysis because of the subjects' medical (diabetes) and medication histories (oral/i.v. corticosteroids and i.v. fluid infusion). The serum potassium level and BSL from most of the remaining subjects were recorded within the 'normal' physiological range (3.7-5.2 mmol/L and 4.0-7.5 mmol/L respectively), except for one subject with a slightly lower serum potassium level and four subjects with elevated BSL, but all were not associated with higher than average albuterol enantiomer levels (Table 6).

## Discussion

This study reflects the variations in the presentation of acute exacerbations of asthma and COPD in a typical emergency department setting, both in disease severity and the treatment required. However, the relationship between dose and plasma/serum level of albuterol appears to be minor (*r^2 ^*≤0.4).

In comparison with some previously reported data [8,9,35], the levels of albuterol enantiomers observed in this study appeared to be considerably higher, particularly among acutely asthmatic patients. In addition, the accumulation of (S)-albuterol and variation in the R:S ratio highlight the need for enantioselective assays when measuring albuterol in a clinical setting.

**Figure 3:**
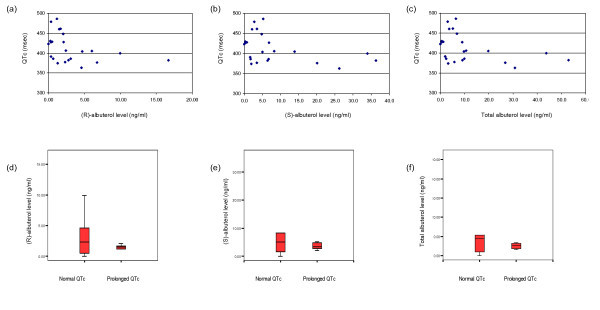
**Relationship between QTc interval and albuterol levels**. Recorded QTc interval and (R)-, (S)- and total albuterol levels and are shown in (**a**), (**b**) and (**c**) respectively. (R)-, (S)- and total albuterol levels in subjects with normal or prolonged QTc interval are shown in (**d**), (**e**) and (**f**).

Recent reviews have raised concerns about the safety of using large doses of β_2_-agonists, especially in patients with underlying cardiovascular comorbidity [27-29]. It has been demonstrated that significant extrapulmonary effects can be observed in subjects given nebulised *rac*-albuterol at a dose of as little as 6.5 mg [9,24,26]. In this study, we observed relatively high albuterol levels in the circulation (some more than 10 times the level observed in the study by Lotvall et al. [24]), but we observed no corresponding variation in extrapulmonary parameters among these patients. The QTc intervals showed minimal change from the commonly regarded normal physiological range, and had no significant relationship with (R)-, (S)- or total albuterol levels (Figure 3). However, evaluation of other metabolic effects of albuterol were more difficult because of complex medication regimens, disease comorbidites, as well as potential psychological (e.g. emotional stress) and physiological (e.g. compensation to respiratory stress) effects. A larger study with greater power may be more helpful to elucidate the other metabolic effects and control for complex medication regimens. The results of this investigation are in line with a previous study that found minimal change in QTc intervals after repeated dosing of a high dose of β_2_-agonist [36]. The findings suggest that the potential extrapulmonary effects of albuterol do not appear to be problematic among patients who use inhaled *rac*-albuterol for the acute relief of shortness of breath, even among patients with underlying cardiovascular comorbidity. However, the long-term effects of accumulation of high concentrations of albuterol enantiomer remain unknown and are the subject of ongoing work.

The wide variation in the relationship between dose and levels has also indicated the difficulties in spot sampling methodology without a population pharmacokinetic model [37], as well as the potential impact from the subject's inhalation technique, particularly when an MDI device is used [38-40].

## Conclusions

High plasma concentrations of albuterol were observed in both asthma and COPD patients presenting to the emergency department. Extrapulmonary cardiac adverse effects (prolonged QTC interval) were not associated with the plasma level of *rac*-albuterol when administered by an inhaler in the emergency department setting. Long-term effect(s) of continuous high circulating albuterol enantiomer concentrations remain unknown, and further investigations are required.

## Consent

Subjects provided written informed consent and the study was approved by the Tasmanian Human Research and Ethics Committee in accordance with the Helsinki Declaration.

## Competing interests

The authors declare that they have no competing interests.

## Authors' contributions

GAJ, RWB, and EHW conceived the study, and participated in its design and coordination. KCY coordinated the study patient recruitment, data collection and undertook the laboratory analysis. KYC and GAJ performed the statistical analysis. All authors helped draft the manuscript. All authors read and approved the final manuscript.
